# Urotropin

**Published:** 1898-02

**Authors:** J. A. Flexner


ARTICLE X.

### UROTROPIN.

BY J. A. FLEXNER, M. D.

The favorable reports that have come to my notice regarding urotropin lead me to think that some additional facts concerning it may not be amiss. Urotropin is a derivative of formic aldehyd, and not a coal-tar product. The well-known antiseptic and preservative power of formic aldehyd in solution led Nicolaier, of Gottingen, to use the solution of formic aldehyd, containing forty per cent. of the gas, and known as formalin, as a means of preserving specimens of urine from decomposition pending examination. He noticed that neither uric acid nor the amorphous urates were deposited, though the same specimens not treated with formalin deposited either or both, as the case might be. Even when already precipitated these substances underwent solution when formic aldehyd was added to the specimen. Urines that readily deposited large amounts of uric acid when acidulated with a mineral acid did not do so when a sufficient amount of the preservative was added.

The results of these investigations prove very conclusively the great uric acid solvent power of formalin The reputation of the lithium salts as uric acid solvents is caused by the common error of applying the results of laboratory experiments to practical therapeutics. The fact that lithium forms an insoluble phosphate, and that the blood and urine contain considerable amounts of soluble phosphates, show that lithium salts, given by the mouth, must form insoluble phosphates of lithium long before they can combine with the uric acid. In this respect it is no exception to the chemical law that when the ingredients for the formation of an insoluble body are present in a mixture this body will always be formed. Medical opinion has for a long time attributed the success attending the use of the lithiated waters to the water and not to the lithium that they contain. A similar error obtains in the case of some more recently introduced substances, lycetol, lysidin, etc. They do not, in the urine, form the very soluble uric acid combinations that they do outside the body. Formic aldehyd being too irritating to be taken internally, Nicolaier then determined to try its amine combination as a substitute. This substance, hexamethylen-tetramin or urotropin is non-poisonous even in considerable quantities, is unirritating, very soluble in water, and is as good a uric acid solvent as formic aldehyd itself.

The name urotropin was given to it on account of the changes which its administration brought about in the urine. Alkalin and putrid urines, containing mucus in excess, pus and pus organisms, uric acid or amorphous urates, were rapidly restored to a normal appearance and an acid reaction. The urine was sterilized and increased in quantity, and calculi and deposits were dissolved. Hence urotropin is a most valuable resource in all suppurations of the urinary tract, and in all gouty and rheumatic conditions where an active eliminant of uric acid and its salts is indicated. A further valuable property of urotropin is its faculty of combining readily with salicylic acid and forming a soluble combination. A solution containing from ten to fifteem grains each of urotropin and salicylic acid to the fluid ounce of water, or other suitable vehicle, has the further advantage over the salicylates alone in that its taste is not disagreeable. It appears to be far less irritant to the gastric mucous membrane than solutions of salicylic acid usually are, and the combination promises to have a wide range of therapeutic usefulness.—*American Practitioner and News.*

